# Embedding new technologies in practice – a normalization process theory study of point of care testing

**DOI:** 10.1186/s12913-016-1834-3

**Published:** 2016-10-19

**Authors:** Caroline H. D. Jones, Margaret Glogowska, Louise Locock, Daniel S. Lasserson

**Affiliations:** 1 Nuffield Department of Primary Care Health Sciences, Radcliffe Observatory Quarter, University of Oxford, Woodstock Road, Oxford, OX2 6GG UK; 2 NIHR Oxford Biomedical Research Centre, John Radcliffe Hospital, Oxford University Hospitals NHS Trust, Headley Way, Oxford, OX3 9DU UK

**Keywords:** Ambulatory care, Case study, Diagnostic tests, Ethnography, Normalization process theory, Point of care technology

## Abstract

**Background:**

Many point of care diagnostic technologies are available which produce results within minutes, and offer the opportunity to deliver acute care out of hospital settings. Increasing access to diagnostics at the point of care could increase the volume and scope of acute ambulatory care. Yet these technologies are not routinely used in many settings. We aimed to explore how point of care testing is used in a setting where it has become ‘normalized’ (embedded in everyday practice), in order to inform future adoption and implementation in other settings. We used normalization process theory to guide our case study approach.

**Methods:**

We used a single case study design, choosing a community based ambulatory care unit where point of care testing is used routinely. A focused ethnographic approach was taken, including non-participant observation of all activities related to point of care testing, and semi-structured interviews, with all clinical staff involved in point of care testing at the unit. Data were analysed thematically, guided by normalization process theory.

**Results:**

Fourteen days of observation and six interviews were completed. Staff had a shared understanding of the purpose, value and benefits of point of care testing, believing it to be integral to the running of the unit. They organised themselves as a team to ensure that point of care testing worked effectively; and one key individual led a change in practice to ensure more consistency and trust in procedures. Staff assessed point of care testing as worthwhile for the unit, their patients, and themselves in terms of job satisfaction and knowledge. Potential barriers to adoption of point of care testing were evident (including lack of trust in the accuracy of some results compared to laboratory testing; and lack of ease of use of some aspects of the equipment); but these did not prevent point of care testing from becoming embedded, because the importance and value attributed to it were so strong.

**Conclusions:**

This case study offers insights into successful adoption of new diagnostic technologies into every day practice. Such analyses may be critical to realising their potential to change processes of care.

**Electronic supplementary material:**

The online version of this article (doi:10.1186/s12913-016-1834-3) contains supplementary material, which is available to authorized users.

## Background

Diagnostic technologies are becoming smaller, lighter and quicker. Many point-of-care (POC) tests are available which are minimally invasive (for example fingerprick tests) and produce results within minutes without the need to send samples to a laboratory [1, 2]. These could be used during clinical visits to enhance prescribing and referral decisions; save time, follow-up visits and costs; and improve patient health outcomes, convenience and satisfaction [3–7]. In particular, they offer the opportunity to risk stratify and deliver acute care in the out of hospital setting, which is an important component of the English National Health Service (NHS) Five Year Forward View [8]. Increasing access to diagnostics at the point of care has also been recognised by the Future Hospital Commission of the Royal College of Physicians to increase the volume and scope of acute ambulatory care [9].

Yet POC tests are not widely used in UK community settings. A recent systematic review of clinicians’ attitudes towards POC testing revealed a number of potential barriers (concerns about accuracy and usefulness; cost, maintenance and time; potential overreliance on tests; patient dislike and anxiety) as well as facilitators to the adoption of these technologies [10]: exploring and addressing these may promote wider adoption and associated benefits.

Comparative case studies of technology adoption/implementation in 12 English NHS Trusts found that consideration of knowledge about how to use the innovation was critical to successful adoption and implementation [11]. Understanding the implementation of POC testing requires exploration of the intervention itself, but also how staff engage with it and adapt to embed it in everyday practice. Studying early adopters of new technology informs other practitioners and providers so they can act as fast followers [12]. We aimed to explore how POC testing is used in a setting where it is embedded and used routinely, in order to inform future adoption and implementation in other settings [13].

This case study was conducted in a community based ambulatory care unit in England, where POC testing is used daily, representing an important opportunity to understand the value and use of POC testing in a routine clinical setting, and to examine in context how POC testing has been implemented. The unit is open seven days a week from morning until evening; and patients are referred by primary care physicians (from the patient’s own practice or an emergency on call service) or ambulance paramedics responding to emergency calls. The unit sees over 300 patients per month that require assessment and interventions that cannot be provided by primary care but does not see patients with life-threatening illness or those in need of an urgent hospital based procedure. It aims to rapidly assess patients with acute medical illness (most of who are older and living with frailty), initiate treatment and support an out of hospital care pathway as much as possible; but if necessary, acute hospital admission is arranged. The doctors are employed by an acute hospital and rotate to the community based unit; and the medical laboratory of the acute hospital provides quality control, support for stock ordering of cartridges for POC testing as well as connectivity of the POC results into a laboratory information management system.

The study was guided by Normalization Process Theory (NPT), a middle range theory (i.e. one which integrates theory with empirical research, to explain observations) developed for examining how new technologies are implemented, embedded and integrated in healthcare settings [14]. We used NPT as a theoretical framework because of its strength in guiding research into how a new technology becomes ‘normalized’ (embedded in everyday practice) within a specific organisation, and identifying factors that promote or inhibit normalization [15]. It can focus on the period when new technologies are normalized and no longer apparent as novel interventions, and is recommended by the Medical Research Council as a tool for understanding how this incorporation into routine practice is achieved [16]. There is growing interest in using NPT to analyse implementation processes in healthcare settings [17–20]. It encourages thinking around how participants interact with each other and with objects and context, and how they work individually and collectively to embed a new technology. We used NPT as a guide to understanding how POC testing is used and has become normalized in the unit under study. NPT is built around four main constructs:

- coherence: how people individually and collectively understand and make sense of implementing a new practice
- cognitive participation: how people build and sustain engagement with the new practice
- collective action: how people enact the new practice
- reflexive monitoring: how people formally and informally appraise and understand the effects of the new practice once it is in use

The constructs influenced our interview topic guide, data analysis and interpretation.

## Methods

This was a case study design, with a single intrinsic case chosen for its routine use of POC testing, which is a unique phenomenon and distinguishes it from other settings [21]. Case studies enable detailed exploration of a phenomenon within its real-world context, and rely on multiple sources of evidence which are integrated in a process of triangulation [21, 22]. We used a focused ethnographic approach for its pragmatic way of examining a specific topic in a discrete organisation using a range of methods including episodic observations with a limited number of participants [23]. All data were collected between October 2014 and February 2015, by one non-clinical researcher (CJ, PhD) who is experienced in qualitative research and a member of the National Institute for Health Research (NIHR) Diagnostic Evidence Co-operative Oxford, with an interest in promoting uptake of POC testing in practice.

A combination of non-participant observation, semi-structured interviews, and document analysis was used, allowing us to focus on the whole range of practices associated with POC testing from the perspectives of participants. For approximately one day per week (on different days of the week), staff at the unit were observed going about their daily tasks relating to POC testing. The researcher overtly wrote anonymous, detailed field notes, and informally discussed with participants what they were doing and why, writing verbatim quotations in the field notes. Hand-written field notes were typed up for analysis. As the researcher was not an accepted member of the clinical team, Wind terms this fieldwork in a clinical setting ‘negotiated interactive observation’ [24]. Additionally, staff were invited to participate in a semi-structured interview to explore and validate observations, using a flexible topic guide developed based on published literature, preliminary findings from the observation, and NPT (see Additional file 1). Interviews took place in a private room at the unit with only the researcher and interviewee present; they lasted up to 50 min, were audio recorded, transcribed verbatim and anonymised. Documents at the unit which were relevant to POC testing were copied, including standard operating procedures (SOPs), training materials and posters, to inform our analysis. Data collection ceased when no further novel themes emerged from new data.

The study was funded by the NIHR Diagnostic Evidence Co-operative Oxford and approved by the University of Oxford Central University Research Ethics Committee (reference MSD-IDREC-C1-2014-113) and the relevant NHS Trust. Written permission for the unit as a whole to participate was obtained from a representative of the unit prior to data collection. All staff at the unit who were involved in POC testing in any way were invited to participate including doctors, nurses and health care assistants (HCAs). The study and its purpose was introduced and explained to staff at a team meeting, through information leaflets and email, and orally by the researcher prior to observation of each participant. Clinical staff had the opportunity to opt out of observation (although none did); and oral informed consent was obtained from all participants. Written informed consent was obtained from all interview participants. Fieldwork was focused on staff not patients; patients were not formally observed, and care was taken to avoid documentation of any information (for example in field notes or interviews) which could identify any patients.

The study design was iterative; initial analyses guided ongoing data collection and analysis, so that the findings were firmly grounded in the data [22]. We focused on all aspects of POC testing, and were guided by NPT, examining the data for each of the main constructs. Data were analysed thematically. Analysis was led by CJ and ongoing analysis was discussed and agreed with MG, a social science researcher with no experience in POC testing and no bias towards its promotion. Through reading and re-reading the field notes and transcripts, a coding scheme was developed collaboratively which included both emergent and pre-identified items. The written data were systematically coded with the assistance of QSR NVivo 10 (a computer software package for qualitative data analysis), and codes were grouped into themes, guided by NPT’s components. We allowed for unexpected issues to emerge, and did not force the data to fit NPT. We used the NPT toolkit [14] to answer questions about how well the data supported each main construct in this case, which produced a radar plot showing the relative strength assigned to each of NPT’s constructs. Although transcripts were not returned to participants for comment, the study findings were presented at a staff meeting to elicit participant feedback, and attendees agreed with the findings and conclusions. Rigour was improved by having multiple sources of data, looking for discrepant data, and discussing and agreeing the codes and themes amongst all authors which included clinical and non-clinical researchers. The findings were also discussed with members of the NIHR Diagnostic Evidence Co-operative Oxford for verification.

## Results

Observation took place on 14 days over 4 months. All eligible participants who were working on those days consented to participate, including doctors, nurses and HCAs. Six participants were interviewed, and the data verified the findings from the observation. At this point no new, rich data relevant to the study were emerging either in interviews or observation, and data collection ceased. One interview was not audio recorded due to participant preference, but detailed notes with verbatim quotes were written.

The findings are presented according to themes, which map onto NPT’s four constructs as shown in Table 1. Data are shown in italics, with verbatim quotations from interviews or informal discussions during observation in quotation marks. Field notes from each observation day were numbered consecutively from 1–14 and interview transcripts were numbered from 1–6, and the numbers are shown for traceability. Details of the POC tests available at the unit are shown in Table 2.

**Table 1:** Main themes and subthemes, and how they map onto NPT’s four constructs

Theme/subtheme	NPT construct
Purpose and value	Coherence
Operationalisation of POC testing	
Deciding which POC tests to take	Collective action
Taking the blood and running the test	Collective action
Examining and acting upon the results	Collective action
Teamwork and communication	Cognitive participation
Physical environment and equipment	Collective action
Learning POC testing	Cognitive participation
Trust in POC testing	Collective action
Appraising POC testing	Reflexive monitoring

**Table 2:** The unit’s monthly usage of POC tests by manufacturer, testing platform and breakdown of individual test components

Manufacturer	Platform	Cartridge (components)	Tests per month
Abbott Point of Care	i-Stat	Chem 8 (sodium, potassium, urea, creatinine, glucose, haemoglobin, ionised calcium, TCO2, anion gap)	240
		CG4+ (pH, PO2, PCO2, TCO2, bicarbonate, base excess, SO2)	5
		PT/INR (Prothrombin time/International normalised ratio)	50
		Troponin	11
Alere	Afinion	CRP (C- reactive protein)	150

### Purpose and value

Staff (doctors, nurses and HCAs) collectively agreed that POC testing is vitally important to the unit; it was referred to as *“crucial… integral to running this service” (Doctor, field notes 10),* and staff frequently commented that they did not know how the unit could operate without it. In fact, POC testing was introduced very soon after the opening of the unit so it did not operate without it for long; and this timing of introduction may have contributed to successful implementation. The importance and purpose of POC testing were linked to the nature of the service: it is an acute unit, open only during daytime hours, therefore needing quick decisions about patient management:

*“Point of Care Testing? I think it’s very important, I don’t think we’d be able to function without it because we’re only open for a short time as well, it means that we can have blood results and patients can be diagnosed almost immediately so it’s really important. I don’t think we could function at all as a unit without it.” (Nurse, interview 5)*

Speed of results is what distinguished POC testing from laboratory testing or no testing for staff, and they collectively understood and appreciated the difference between POC testing and other ways of working in this respect. Speed of results enabled quick decisions, including diagnosis, treatment and referral:

*The nurse described using the POC test results to make an initial treatment plan… They also make decisions to refer patients to hospital based on abnormal POC test results. It’s about “safety” and if it’s safe to send patients home, or if they need to be monitored/treated. They also make treatment decisions based on POC test results, for example what amount of antibiotics patients need. (Extract from field notes 2)*

Participants recognised that POC testing is valuable in community settings, and when quick decisions are needed; but were less certain about the purpose of POC testing in acute hospitals if laboratory test results are available quickly, or other in-patient settings where quick decisions about whether it is safe to send a patient home may not be required.

### Operationalisation of POC testing

The daily tasks involved in carrying out POC testing were deciding which tests (if any) to take for each patient when they arrived; communicating this to others; taking the blood; running the tests; examining the results; communicating the results to others; and deciding what action to take accordingly. This work was allocated to different staff according to their skills and availability. Close teamwork appeared key to ensuring that each task was performed by an appropriate person at the necessary time.

#### Deciding which POC tests to take

There were a number of different POC tests available including a clinical chemistry profile (electrolytes, renal function), haemoglobin, C-reactive protein (CRP), troponin, blood gases (including lactate), and International Normalised Ratio (Table 2). The POC testing format involves blood samples inserted into cartridges which are then analysed by the reader, which is either handheld or a small bench top unit. Deciding which POC tests to take for each patient was primarily the responsibility of the doctors on duty, according to *“clinical need… indication” (Doctor, field notes 10),* and decisions were communicated to nurses and HCAs verbally and by written notes. Some nurses also collaborated with doctors to make a decision, or made some decisions alone. Some, but not all, nurses described that they had learned which POC tests are needed for different types of patient presentations, so they were confident to suggest to the doctor which might be needed, or make that decision alone if a doctor was not immediately available to ask. This indicates increased knowledge and learning for nurses through POC testing (see below).

#### Taking the blood and running the tests

Taking the blood and running the tests was done by nurses and HCAs. Blood for both POC testing and laboratory testing was taken at the same time. Staff knew that the blood for POC testing needed to be taken first and that POC tests must be run within as short a time as possible after the blood was taken, and they managed this by working in pairs:

*“what we tend to do here is we try and work in pairs so if we need to take lab bloods as well, we take [POC testing] bloods first and ask somebody to go and run the [POC tests]… and then the other person taking the blood will carry on taking it, so it’s kind of, it’s as quick as we can do it really (Nurse, interview 3)”*

*The nurse took blood in different syringes/tubes – then she called “is anyone free to do a [POC] test?”; a HCA straight away said yes, came and collected a syringe of blood (Extract from field notes 6)*

Close teamwork enabled staff to call on others for help when needed.

#### Examining and acting upon the results

POC test results were printed or written down; and for some markers normal ranges were available so nurses and HCAs could identify any abnormal results and mark these with pen. Results were always passed to a doctor. However some nurses began to interpret the results and communicate this to the doctor:

*the nurse said “CRP is 133, I think she’s got a urine infection”… The doctor said “OK”. (Extract from field notes 7)*

*“so we can kind of half come up with a plan really and then the Doctors, you know, see them afterwards… so it works for us… I mean you can sort of look at something and think “Well their creatinine and urea is sky high, they’re going to need fluids” or you know, so you can say, go up to the Doctor and say “Actually you know, they’re really dehydrated” and they’ll prescribe the fluids, so it’s kind of working in a team with the Doctors as well, we’re all quite close, you know, not just the nursing and HCA sort of staff, it’s with the medics as well, yeah, and they sort of trust our role, you know, what we do and our judgement” (Nurse, interview 3)*

#### Teamwork and communication

Close teamwork and trust amongst all levels of staff were strongly evident in operationalising POC testing, and acknowledged by staff who described the team as *“not hierarchical”, “a lovely team… ever so supportive” (HCAs, field notes 14 and interview 1)*.

Tied to close teamwork and effective operationalisation of POC testing was good communication. Formal methods existed for communicating about POC testing including daily team meetings, patient notes, and a large white board with information about each patient. Written notation on the board was not always consistent (for example different terms were used); but regular informal communication overcame any limitations and ensured that all staff were aware of what was happening around POC testing:

*“I think we’ve got good communication and we kind of know who’s [POC test] is in when” (Nurse, interview 3)*

*“another good thing we do here, everybody is aware of our [POC test] results” (HCA, interview 1)*

#### Physical environment and equipment

A factor which facilitated operationalisation of POC testing was the layout of the centre. Equipment was stored in a clinical room a very short distance from the patient bays so blood could be taken there and tested very quickly (which is important because POC tests are intended to be carried out immediately once the blood is taken). The open-plan nature of the centre facilitated communication amongst all staff. In the clinical room there were posters and information leaflets on display to remind staff of POC testing procedures.

Some factors related to equipment set-up were potential barriers to POC testing. Staff barcodes were not recognised when scanned, and patients’ NHS numbers could not be scanned, hence staff were required to enter these numbers manually, which was fiddly, time consuming, and meant that they had to find a sticky label with the patients’ NHS number on:

*She scanned her staff card barcode, and it said error, and she pressed continue. She said it does that every time…She then manually entered the patients’ NHS number from the patient’s records. (Extract from field notes 2)*

*“The only thing I think with that is, if you haven’t got the sticky label and the numbers on it and then they’re saying “we need to do the blood test as quickly as you can”, so that it doesn’t affect the results, if you then are kind of waiting around and trying to find a sticky label or something, can be a delay in trying to do it.” (Nurse, interview 6)*

Despite these complications, staff remained overwhelmingly positive about POC testing:

*“that doesn’t bother me at all, it’s just time consuming, you know, or if you press the wrong button and it all disappears and you, and you’ve got to start again! But no, that’s not too bad.” (HCA, interview 1)*

The only criticism that staff had was the number of machines being insufficient, and causing queues during busy periods. The equipment was described as easy to use, once the technique (particularly inserting blood into the testing cartridges) had been learnt.

#### Learning POC testing

In order to learn POC testing, new nurses and HCAs watched others perform POC testing, then had others supervise them to ensure they were doing it correctly. High staff turnover and the associated need for regular training was acknowledged as an issue for maintaining consistently high-quality practice amongst all staff.

Several participants described that since the introduction of POC testing there had been some retraining, initiated by a member of staff who felt that POC test results were not always trusted, and that more consistency was needed:

*“one of the [nurses], she sort of took it on and sort of thought, “right, okay, I want to know that this is being done properly”” (HCA, interview 1)*

She wrote a Standard Operating Procedure (SOP) and a quiz for staff, to ensure that they understood the optimum procedures for POC testing (including taking the sample, processing the blood and performing the test, storing and maintaining the equipment). Staff felt that practice had changed, and become more consistent, in line with the SOP; specifically: 1. blood for POC testing is now usually taken in heparinised syringes and constantly rolled until testing to prevent clotting; 2. blood for POC testing is taken prior to sampling for laboratory testing, and very shortly after the tourniquet is applied; 3. POC testing is performed as soon as possible after the blood is taken; and 4. POC tests are performed in the optimum order.

#### Trust in POC testing

The introduction of the SOP, and improved consistency, was thought to improve trust in POC testing and results, because *“consistency equals quality doesn’t it” (Nurse, field notes 8)*. Trust in POC testing was complex. Staff described trusting the results as long as the blood was collected and tested appropriately:

*“if you don’t get a good flow then the results are not going to be accurate… sometimes the doctors have said “can you get me another specimen because I’m not convinced that that is accurate” so we’ve had to do it again and sure enough, the potassium levels have been lower and so it’s obviously the way the blood was taken or the length of time before it got in to the machine so you’ve got to understand the importance of doing it properly” (HCA, interview 1)*

Despite introduction of the SOP increasing trust in POC testing, there was still some feeling that POC testing is not as accurate as laboratory testing:

*The doctor said POC testing is very very useful, and that “it’s accurate in inverted commas, not lab sample accurate” – I probed, and s/he said it can’t be as accurate as lab testing, and if you send the same blood to the laboratory and for POC testing there will be slightly different values… I asked how s/he knows it’s inaccurate – s/he said because doctors and nurses say that, and also observation of his/her own. (Extract from field notes 10)*

It was commonly mentioned that results for three particular biomarkers were inaccurate compared to laboratory values; and staff had learned how to interpret the results, for example at what level the results are truly abnormal. Generally it was felt that the results are accurate enough for their purpose of enabling quick decisions:

*“Yeah, it is pretty accurate, you can sort of see when the lab results come back the next day or in the evening, sort of compare it, it can be slightly off but nothing that would alter your treatment” (Nurse, interview 3)*

However for various reasons, including limited trust and confidence in POC testing, markers tested by POC tests were often also tested in the laboratory. Lack of confidence that POC test results would be seen by the appropriate people, due to not consistently being linked up to the same systems as laboratory results, also led to POC tests being repeated as laboratory tests. Laboratory testing was also conducted alongside POC testing because laboratory testing offers a much wider range of available tests. Although at first there appears to be unnecessary repetition between POC testing and laboratory testing, closer analysis revealed that staff see POC testing and laboratory testing as working together, and having different roles:

*“do like to have the labs as well just to compare them.. to look at cumulative I think and so we can keep track on our case notes and on the lab results, they can print off cumulative results which are quite useful for our patients that are coming in sort of daily and stuff… but for our decision making at the time and to provide a plan for that patient and give them the best treatment we need to have that [POC test] result in our hand there and then kind of thing…I think there’s still a value in lab bloods, obviously there’s so many tests through lab… lab bloods it’s just more specialised and I think there’s always going to be a place for that… and I think, you know, there’s things that you need to know immediately and there’s things that you don’t, and I think that’s the difference, yeah” (Nurse, interview 3)*

### Appraising POC testing

It was as a result of appraising POC testing procedures and believing them to be inconsistent that one nurse introduced a SOP and quiz to ensure that POC testing was being performed consistently (see above). Staff now individually and collectively appraised POC testing as worthwhile, using informal methods (such as discussing amongst themselves the value of POC testing) and formal methods (for example information on patient management – including length of time patients spend in the unit, readmission and referral rates – was displayed on notice boards in the unit, and staff felt that POC testing contributed to their success in these areas). Although the accuracy of some POC tests compared to laboratory tests was questioned, the recognition that this does not usually change patient management led to POC testing being appraised as worthwhile.

Whilst all staff acknowledged the benefits of POC testing for patient care, some felt that POC testing was not immediately apparent to patients, whilst others thought that patients were surprised and happy to be offered POC testing and benefitted from increased reassurance:

*“I think it [POC testing] reassures the patient that actually the bloods show that there’s some sort of infection there or inflammatory response and they sort of, obviously there’s a lot of stuff about antibiotics at the minute, about whether we should be using them so I think it just gives them that bit of a back-up, a bit more confidence in why we’re making that decision… I think it does provide a better experience for patients, it’s smoother and yeah, they obviously get their treatment quicker” (Nurse, interview 3)*

The high cost of POC testing was recognised, but participants considered it to be financially worthwhile:

*“The [POC test] cartridges are quite expensive, but worth it, because they can keep patients out of hospital” (Nurse, interview 5)*

A potentially negative impact of POC testing that occurred rarely in the data was the perception that POC tests might be overused, with patients receiving tests that they do not need, or the results being relied upon too heavily; however this was not explicitly described as a negative impact:

*The doctor said a danger is if there’s a test available it becomes THE definitive test for something, but it isn’t; for example some patients here don’t have high CRP, but they clearly have an infection; so the test is just part of the picture. (Extract from field notes 14)*

*“being over-used maybe, but I don’t know, is that a bad thing or not if they pick up extra problems that maybe someone didn’t know that was wrong and pick up extra things.” (Nurse, interview 6)*

Further to being worthwhile for patients, the running of the unit, and financially, a perhaps unanticipated benefit of POC testing was that it increased skills, knowledge and job satisfaction for nurses and HCAs, who said they *“love doing it”* and felt *“privileged” (HCAs, field notes 7 and interview 1)* to do so:

*“seeing it from my point of view, if I can see something [a POC test result] that’s abnormal then I can ask questions and I want to know why, what does that mean, you know, and that’s a big thing for me, rather than just being told “take those bloods” and not know why you’re doing it, you know.” (HCA, interview 1)*

*“it’s definitely sort of made me more aware of normal ranges and you can kind of look through a printout [of POC test results]… and you know sort of instantly know if there’s something that stands out… I think it’s knowing, you know, the rationale behind using them [POC tests] that’s, you know, another element to it, it’s not just taking blood, there’s a lot more to it and I think it, you know, it’s an enhancer, it’s your assessment skills as well because you’re looking at these results and, you know, it sort of prompts you really to act upon them or, you know, kind of think why is that skewed? Why is that deranged? You know, is that accurate? Should I recheck it? Or should we send labs and then, you know, wait for those to come back? Is it something that we can act on now?” (Nurse, interview 3)*

### How POC testing has become embedded

**Fig. 1:**
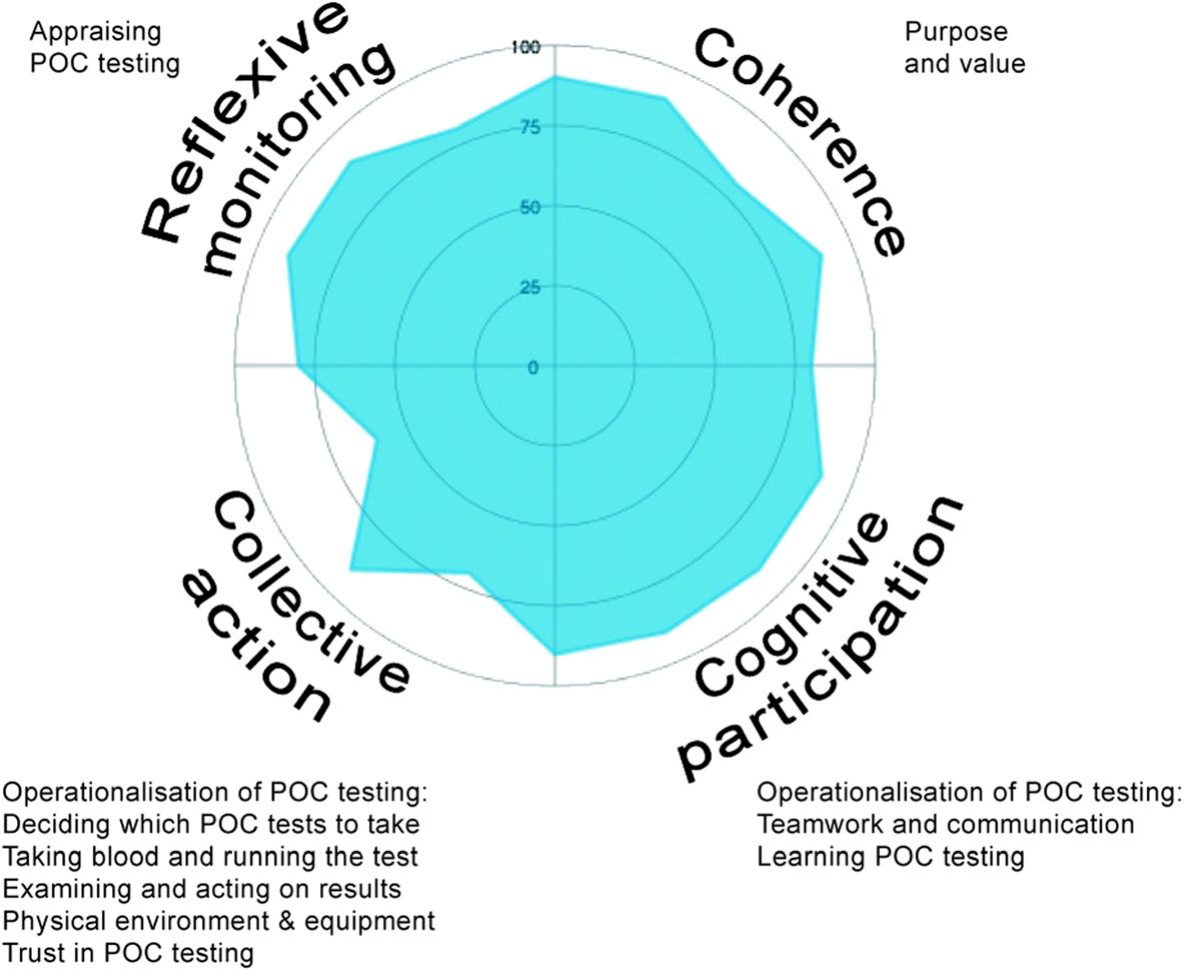
Radar plot showing the extent to which the data supports each construct of NPT. Closer proximity of the shaded area to the outer line of the circle indicates greater strength, and closer proximity of the shaded area to the centre of the circle represents lesser strength, assigned to that construct

It was very evident that POC testing is embedded in the unit and a routine part of everyday working. Applying NPT enables us to examine how it has become embedded. Figure 1 shows the extent to which the data supported each of NPT’s constructs in this case study. It was created by answering questions on the NPT toolkit about how well each main construct was established, based on the data [14]. The figure highlights the relative strength assigned to the coherence, cognitive participation and reflexive monitoring constructs. Overall, staff understood and appreciated how POC testing differs to laboratory testing and had a shared understanding of its purpose, value and benefits (coherence); they organised themselves as a team to ensure POC testing worked optimally, with everyone supporting it, and one key individual drove POC testing forward by introducing a SOP to improve consistency (cognitive participation). Staff all assessed POC testing as worthwhile for themselves, patients, and their unit; and it was as a result of monitoring that the SOP was introduced to improve consistency (reflexive monitoring). The collective action construct of NPT is not so strongly supported: although tasks were appropriately allocated to doctors, nurses and HCAs, and they effectively operationalised POC testing by working in pairs and following the information in the SOP, comparisons between laboratory test results and POC test results led to staff trusting the accuracy of some POC test results more than others; and there were some issues with the equipment such as staff and patient NHS numbers needing to be entered manually, which could cause delays and potential errors. However these potential barriers did not prevent POC testing from becoming embedded, because the coherence, cognitive participation and reflexive monitoring constructs are so strongly supported: staff collectively and individually understood the purpose and value that POC testing added, and there was a strong belief that the unit could not operate effectively without it.

## Discussion

This case study provides detailed information on factors leading to successful adoption of POC testing, which can be transferred to improve implementation in other settings [11]. In addition to gaining practical information about using the equipment, it emerged that close teamwork, trust and good communication amongst all staff were key in the normalization of POC testing. Using NPT strengthened our analysis and interpretation, uncovering how doctors, nurses and HCAs established and maintained strong coherence and participation in POC testing, and appraised its worth and made adjustments to improve its value. The sense of value, purpose and importance attributed to POC testing, which was perceived to be integral to the aims and running of the unit, overcame any potential barriers and ensured that it was normalized despite difficulties such as entering information manually into the equipment. However, commissioners and industry could consider how seemingly little things, such as the need to enter barcode numbers manually, could impact on the process of adoption, and on participants’ trust in the technology and the results it provides. Adoption could be supported further by ensuring that staff are convinced about the accuracy of POC testing, by collecting and presenting appropriate evidence. In this unit, despite limited trust in some of the POC test results, doctors had learned how to interpret and act on results accordingly, because the value of doing so was so well established and accepted.

Research into POC testing has focused on accuracy, patient outcomes, antibiotic prescriptions, cost effectiveness and increased job satisfaction [5–7, 25, 26]; but this study has uncovered another potential benefit in the form of an increase in nursing and HCA staff knowledge about disease and critical laboratory reference ranges.

Although POC testing was successfully embedded in this unit, the data illustrate potential negative impacts of POC testing to be addressed. Potential overuse or overreliance on POC testing was mentioned (although this did not emerge strongly), as highlighted previously [27, 28]. Whilst not supported by our data, the potential for over-testing is important in the context of over-diagnosis and its iatrogenic consequences, and potential for increased patient expectations, inappropriate investigations and referral [29]. Although staff perceived POC and laboratory testing to serve different functions, some POC tests were repeated in the laboratory (for reasons including traceability and accuracy of results), suggesting that in the future clear guidance may be needed on when to use laboratory testing only, POC testing only, or both types of testing. The data suggest that patients who are aware of POC testing are reassured and provided a better experience. Further research planned by the authors will explore patients’ experiences in this unit in detail, since the perspectives of all stakeholders are important when analysing implementation of new practices [17].

The strengths of our study include the multiple sources of data, with interview data verifying findings from non-participant observation; organising fieldwork to take place at different times of day and days of the week; the 100 % response rate for observation; and analysis and interpretation being discussed and agreed amongst a team of researchers including clinical and non-clinical, and those with and without a particular interest in promoting POC testing. While the nature of case studies means that generalisability cannot be determined, the use of NPT as an explanatory framework provides transferable understanding of how POC testing can become normalized, which can be applied to other settings. Of course, implementing the same POC tests in a different setting will not mean that they will be adopted or embedded in the same way; the four NPT constructs may play out differently in different settings with different staff [20, 30]. Particularly, while POC testing is being designed increasingly with low-resource settings in mind, the experience of implementing it in these settings is likely to be different from its implementation in developed countries [30].

Future research should explore the adoption of POC testing in other settings, and from the perspectives of other stakeholders including patients, carers and managers, as well as continuing to examine the clinical- and cost-effectiveness and impact on patient outcomes.

## Conclusion

Whilst there are many barriers to the diffusion of innovation within healthcare settings, this study of healthcare professionals’ experiences of new diagnostic technologies offers new insights into successful adoption. Such analyses may be critical to releasing the potential of new diagnostic technology to change processes of care.

## Additional file

Additional file 1: Interview Topic Guide. Flexible interview topic guide which was used to guide interviews. (DOCX 18 kb)

## Abbreviations

CRP: C-reactive protein
HCA: Health care assistant
NHS: National health service
NIHR: National Institute for Health Research
NPT: Normalization process theory
POC: Point-of-care
SOP: Standard operating procedure
