# Supplementary material for: Embedding new technologies in practice – a normalization process theory study of point of care testing
Source: BMC Health Serv Res. 2016 Oct 19;16:591. doi: 10.1186/s12913-016-1834-3 (PMC5070078; doi:10.1186/s12913-016-1834-3)
Supplement: Additional file 1: — Interview Topic Guide. Flexible interview topic guide which was used to guide interviews. (DOCX 18 kb) [file 12913_2016_1834_MOESM1_ESM.docx]

# Flexible Interview Topic Guide

(Please note this was a flexible topic guide for guidance and was not rigidly adhered to)

*Using point-of-care tests*

- Can you describe how point-of-care testing is used here?
- How do you use point-of-care testing here (e.g. order/ take tests, interpret results)
- Who is responsible for each aspect of point-of-care testing?
- When and where are the tests taken?
- How do you find the process of taking point-of-care tests?
- Which patients receive which point-of-care tests? (Who decides which tests each patient has?)
- How are the results processed?

(Who looks at the results and when? How are the results interpreted and who by?)

- (How) did you learn to interpret the results?

(Did you have any difficulties interpreting the results, and how did you resolve these? What do you do when you have intermediate results?)

- How important is point-of-care testing here?
- Do you have any experience of using point-of-care testing at other centres/units?

*Learning to use point-of-care tests*

- How did you learn to take point-of-care tests?
- How was the process of learning? (How easy was it to learn and how long did it take?)

*Equipment*

- Have you had any difficulties using the equipment? (What were they, what did you do?)
- Where is the equipment kept?

(Is this space appropriate? What space is needed to store and use equipment?)

- How is the equipment maintained?

(What is done to maintain the equipment? Who is responsible for maintaining and checking the equipment, and who does this? How often?)

*Patient attitudes to the test*

- How do patients react to having point-of-care tests taken?

*Impact of point-of-care testing on clinical practice*

- Do you think that having point-of-care tests alters diagnostic decision making?

(How? In what situations? Has the process of decision making changed? Are you more or less confident in your decisions? Do you use the test to rule in or rule out diagnoses?)

- Do you think that the availability of point-of-care testing has had an impact on referrals?

(Do you refer more or fewer patients? Do you refer patients to the same place/same speed?)

- (How) do you think point-of-care testing has impacted clinical practice?

*How point-of-care testing has become normalised/ embedded in everyday practice*

- What did you think about point-of-care testing when you first began to work here?
- How did you feel point-of-care testing related to previous work practices?
- What would the differences be if point-of-care testing was not used here?
- How does the availability of point-of-care testing impact the way the unit works?

(e.g. Are fewer laboratory tests ordered?)

- Is it clear who is responsible for each aspect of point-of-care testing?

(Ordering/ taking/ interpreting point-of-care tests)

- How do staff interact and communicate about point-of-care testing?
- How confident are you/ the unit in point-of-care testing?
- What policies/ protocols/ procedures are in place for point-of-care testing?
- How effective and useful does the unit think point-of-care testing is, and why?
- How effective and useful do you think point-of-care testing is, and why?
- How does the availability of point-of-care testing impact other areas of your work/workload?
- What is the value of point-of-care testing?
- (How) have the procedures/practices around point-of-care testing changed over time?

*Future use of point-of-care testing*

- Do you think point-of-care tests should be implemented more widely?

(Could they be used routinely in other settings? Which settings/patients would point-of-care testing be most useful for?)

- How easy do you think it would be to implement point-of-care tests more widely?
- What advice would you have for other organisations wishing to implement point-of-care testing?

*Any other remarks?*

- Is there anything else you’d like to discuss?
